# Modified team-based and blended learning perception: a cohort study among medical students at King Saud University

**DOI:** 10.1186/s12909-021-02639-2

**Published:** 2021-04-08

**Authors:** Ahmed I. Albarrak, Nasriah Zakaria, Jwaher Almulhem, Samina A. Khan, Norshahriza Abdul Karim

**Affiliations:** 1 Medical Informatics and E-Learning Unit, Medical Education Department, Health Informatics and Promotion Research Chair, College of Medicine, King Saud University, Riyadh, Saudi Arabia; 2 UM eHealth Unit, Faculty of Medicine, University of Malaya, Kuala Lumpur, Malaysia; 3 College of Computer and Information Science, Prince Sultan University, Riyadh, Saudi Arabia

**Keywords:** Blended learning, Medical informatics, Medical education, Modified team-based learning flipped classroom

## Abstract

**Background & Objective:**

Medical schools have evolved toward competency-based education and active learner-centered strategies. Medical informatics course was introduced in 2011 in the 3rd year at the College of Medicine (CoM), King Saud University (KSU), to enhance future medical graduates with technological and information competencies. Modified team-based learning and blended learning were emphasized using face-to-face lectures, various e-learning technologies, workshop and seminars. The current study’s main objective was to assess students’ perceptions towards blended and modified team-based learning at the CoM in KSU.

**Methods:**

A survey was distributed to medical students in three consecutive years: 2017–2019. The survey contains items regarding student perception of various types of blended learning techniques applied in the course. The survey was administered using *i-Clicker*; an interactive device that enables students to answer survey questions. Descriptive statistics were used to examine the perception of students on these blended learning dimensions investigated.

**Results:**

Seven-hundred and one student responded to the questionnaire (male; 69.5%, female 30.5%). Out of which, 59.1% of students found team interactions positively supported discussions and asked questions freely, and 48.1% expressed that working in groups facilitated their learning process. However, 56.0% of students chose face-to-face lectures as the most preferred class activities followed by discussion 23.8%. More than 78% of participants agree that online quizzes are good experience and enjoyable. Grade center where students can check for marks and attendance also received high perception (66.3%).

**Conclusion:**

Introducing modified team-based and blended-learning are considered challenging, and therefore, investigating their perceptions can provide useful insights into how these methods could be used more effectively. The blended-learning technique is highly essential in teaching medical informatics to overcome challenges faced due to a large number of students and the need for various exposures to reach the course’s learning goals. Moreover, it is noticed that students were engaged in face-to-face and online activities, furthermore, modified team-based learning reported facilitating learning and asking questions without embarrassment.

**Supplementary Information:**

The online version contains supplementary material available at 10.1186/s12909-021-02639-2.

## Background

Effective medical education is essential for better health care and it is essential element to improve patients healthcare outcomes [1]. However, traditional lecturing, has been blamed for the undesirable effects [2] including; low efficiency and negative learning outcomes [3–5]. Many approaches have been adopted in medical education to help improving students’ skills such as problem-based leering, Team-Based Learning (TBL), e-learning, flipped classroom, blended learning, and case-based teaching. E-learning as a platform has grown noticeably in medical education to ensure self-directed learning opportunities [6]. Research evidence shows that e-learning can be as effective as traditional learning and help learners to gain new knowledge and skills [7]. E-learning offers learners better control over the content, learning sequence, pace of learning and time, providing them opportunities to tailor their experiences to their own personalized needs [8]. However, significant weaknesses encountered with only e-learning included reduced social interactions, limitations of practices, loss of teacher-student relationship and absence of evaluation methods [9]. Therefore, instead of adopting a completely e-learning approach, a blended learning combines both e-learning technology with traditional education, which merges the strengths of both approached [10–12]. A recent study found that Learning Management System (LMS) introduction was associated with improved attendance of face-to-face lectures. Also blended learning groups achieved higher formative test scores and higher summative grades than the traditional group [13].

Active learning in medical education is more effective than passive learning. TBL is an active-learning and group-based instructional format, were first introduced in 1994 [14]. Many medical schools adopted TBL in the preclinical, the clinical, and the residency curriculums, [15]. TBL has become increasingly popular in medical education because of the importance of team management in providing quality patient care [16].

At King Saud University (KSU), Saudi Arabia, e-learning deanship was established in 2009. Blackboard (BB) as LMS was provided. In 2011, Medical Informatics course was introduced into the third-year undergraduate medical curriculum with two credit hours. The course was redesigned later in 2016 to incorporate modified TBL and modified flipped class concepts. The course involves several components including face-to-face lectures, recorded lectures, workshops, and modified flipped classroom.

Therefore, investigating students’ perceptions towards modified TBL and blended learning conducted during the course can provide useful insights into these approaches and can be used effectively in other courses and with other students’ groups. This research study is highly essential in providing insights into how blended learning and modified TBL is perceived among medical students. The current study’s main objective was to investigate students’ perceptions towards blended and modified TBL as practiced at the CoM.

## Methodology

### Study design and instrument

This cohort study used a self-administered structured questionnaire in the English language and adapted from previous work [16–18]. The survey included 27 validated questions categorized into two main sections: 1) Blended Learning approach and its experience (22 questions), and 2) Modified Team-based learning outcomes (3 questions). The remaining two questions were general. These questions were designed as categorical (3 items) and Likert-scale (22 items).

### Setting and subjects

The study was conducted in 3 years on students of 3rd-year medical students in the year 2017, 2018, and 2019 respectively, at the CoM, KSU, Riyadh, Saudi Arabia.

### Medical informatics course

In 2011, a medical informatics course for undergraduate medical students was introduced in the CoM, KSU. The Medical Informatics course was integrated into the third-year undergraduate medical curriculum and was assigned two credit hours. It was redesigned later in 2016 by integrating several components, face-to-face lectures, recorded lectures, workshops, educational resources, and tutorials.

A modified flipped class approach [19–21] and modified TBL was introduced. The first component is the face-to-face lectures where some topics were delivered, then lecture slides are uploaded with other course resources on BB, which is the LMS. The second component is recorded lectures, which had been delivered by using virtual classes on BB. Third component is tutorials where the modified flipped class approach and modified TBL were implemented. Fourth component is workshops which was applied for more of students’ interactions and engagement and practicing problem-solving skills. The two workshops were three-hour workshops. The first workshop was medical error predesigned cases given to students and they were asked to analyze the case assigned to their group. The second workshop was paper appraisal workshop where assigned medical informatics scientific articles were given to students and they asked to answer structured set of questions. Furthermore, modified flipped class approach and modified TBL were applied in workshops.

Modified TBL were implemented based on four principles clarified by Michaelsen (2004) [22]. Proper group formation was implemented during orientation lecture where students divided themselves into groups between four and five students per group who remained as a group for the entire semester and they attend all course activities. Student accountability was performed through asking students to read all the educational resources uploaded in BB before joining classes. Structured assignments were posted on BB one week before the tutorials for each group which necessitate team interaction. In every tutorial session, two groups were presenting for the assignments which is related to the same topic. However, all groups present in the same class and encouraged to participate in the discussion. Each group is supposed to deliver a 15 to 20 min presentation. Last principle is instructor feedback which provided immediately and frequently after solving group assignments. In paper appraisal workshop, students individually solved quiz using *i-Clicker* which assess their understanding of the presented articles. Students were graded based on these activities. Only readiness assurance which involve some testing tools like TRAT and IRAT, which is one of the modified TBL elements [22] is not implemented during this course due to large number of the students per class. However, three online quizzes, which were solved individually, were given to students during the course to assess their understanding regrading several topics.

### Data collection

The study was conducted during a mandatory activity called paper appraisal workshop at the end of every semester for the medical informatics course. This activity applied modified TBLby grouping every ten students in a group and assigning a research paper to read, summarize, and present at the given time slot. Students’ responses were collected using *iClicker* [23, 24], a Student Response Systems; a Classroom Engagement Tool.

### Ethics statement

All participants were informed about the study’s aim and their consent for participation was recorded at the beginning of the study. In addition to departmental approval, the research study was scientifically approved by the Institutional Review Board [Ref. No: E-20-4765], Health Sciences Collages Research on Human Subjects, Research Center, Deanship of Scientific Research, KSU, Riyadh, Saudi Arabia and with accordance with the declaration of Helsinki for research involving human participants or human material and Human Studies.

### Data analysis

The collected data exported in Excel File format from the *iClicker* and was imported using standardized entry codes into Statistical Package for Social Sciences (SPSS). For all tests, statistical significance was set at *P* < .05. Descriptive statistics were used to present means, SD, and percentage. Descriptive statistics were used to examine the perception of students on these blended learning dimensions investigated. T-Test was employed to investigate the differences in gender and Analysis of Variance (ANOVA) for statistical differences between the years in which the questionnaire was administered. For all tests, statistical significance was set at *P* < .05.

## Results

### Response rate and characteristics of participants

Out of 821 students (266, the year 2017), (249, the year 2018), and (306, the year 2019) approached, a total of 701 responded, giving a response rate of 85.3%. Four-hundred and eighty-seven were males (487/701, 69.5%), and 214 (30.5%) were female students. The highest number of students was from the academic year of 2019 (285/701, 40.7%). Table 1 shows the response per academic year.

**Table 1:** Students responses per academic year for both male and female students

		Year			Total
		2017 (***n*** = 266)	2018 (***n*** = 249)	2019 (***n*** = 306)	
**Gender**	**Male**	139 (52.2%)	176 (70.6%)	172 (56.2%)	487 (69.5%)
	**Female**	101 (37.9%)	0 (0%)	113 (36.9%)	214 (30.5%)
**Total (%)**		240 (34.2%)	176 (25.1%)	285 (40.7%)	**701 (100%)**

### Modified team-based learning

In total, three hundred and seventy-two students (56%) prefer “face-to-face lectures” as a useful academic activity, followed by “discussions” (158/701, 22.5%). Students on activities found tutorials (158/701, 22.5%) and workshops (146/701, 20.8%) helpful learning activities and they (agreed strongly and agreed) that working as a team (Tutorials: 53.3%; Workshops: 44.2%) have facilitated their learning process. More than half (362/701, 51.6%) of participated students coincide that team interactions in modified TBL activities like tutorials/workshops have conceded them to ask questions without feeling embarrassed. (See Table 2 for details).

**Table 2:** students’ response for modified TBL elements

	Item	Agree (n%)	Disagree (n%)	Undecided (n%)
1	Lecture is a helpful learning activity	339 (48.36%)	190 (27.10%)	119 (16.98%)
2	Oral explanations in lectures helped to understand textbook chapters	310 (44.22%)	226 (32.24%)	135 (19.26%)
3	I find weekly tutorial a helpful learning activity	158 (22.54%)	403 (57.49%)	94 (13.41%)
4	Working in a group for tutorials facilitated the learning process	374 (53.35%)	183 (26.11%)	86 (12.27%)
5	I find workshops helpful in learning activities	146 (20.83%)	383 (54.64%)	125 (17.83%)
6	Working in a group for workshops facilitated the learning process	310 (44.22%)	253 (36.09%)	82 (11.70%)
7	Reading, analyzing and writing a report helped to improve my analytical and writing skills	208 (29.67%)	232 (33.10%)	147 (20.97%)
8	Team interactions in tutorials/workshops allowed me to ask questions without feeling embarrassed	362 (51.64%)	101 (14.41%)	149 (21.26%)
9	The continuous oral and written feedback from course instructors through different blended learning channels was useful in my learning	382 (54.49%)	105 (14.98%)	171 (24.39%)
10	The class evaluation of presentations was effective	357 (50.93%)	134 (19.12%)	146 (20.83%)

### Blended learning: blackboard (BB)

Students were asked to evaluate their experience with the CoM’s learning management system; Blackboard (BB), a tool of Blended learning approach [25, 26], used in the study. More than half of students (53.1%) found BB user friendly. They reported that using BB for downloading lectures, reading materials, and uploading assignments (264/701, 37.6%), and taking online quizzes (521/701, 74.3%), and E-attendance (492/701, 70.2) %, have facilitated their learning process. They further coincide with these features as useful time saving and enjoyable learning activity experience. Table 3 shows students’ views on (BB) features.

**Table 3:** Students’ views on black board features

	Never (n%)	Barely (n%)	Sometimes (n%)	Frequently (n%)	Always (n%)
I receive emails and read announcements from the Blackboard for course updates.	97 (13.84%)	94 (13.41%)	107 (15.26%)	123 (17.55%)	219 (31.24%)
I download content (slides, chapters, and papers) on Blackboard.	184 (26.25%)	119 (16.98%)	92 (13.12%)	80 (11.41%)	166 (23.68%)
I check and receive grades via Grade center on Blackboard.	92 (13.12%)	23 (3.28%)	36 (5.14%)	68 (9.70%)	430 (61.34%)
I access the Saudi digital library through Blackboard.	206 (29.39%)	91 (12.98%)	109 (15.55%)	97 (13.84%)	169 (24.11%)
I would be interested in using Blackboard in future training that is related to my medical education.	134 (19.12%)	135 (19.26%)	148 (21.11%)	111 (15.83%)	101 (14.41%)

#### General BB usage

Four hundred and thirty students (61.3%) showed that they ‘always’ use BB to check and receive grades via Grade center, following (123/701, 17.5%) ‘Frequently’ to receive emails and read announcements/updates from the course. A small number of students ‘barely’ (119/701, 16.9%) used BB to download learning materials content from BB. In particular, to access the Saudi Digital Library (SDL). Students responded to the question about the most useful tool on the BB, (252/701, 36.0%) voted “Grade Center” most useful tool followed by “Online Quizzes” (217/701, 31.0%). It was observed that the BB feature of “Online Quizzes” was the most preferable/useful tool in 2017. The tool “Grade center” took most votes in 2018 and again was most voted in the year 2019, with no significant differences between year and the types of BB tools used (see Table 4).

**Table 4:** Students responses per academic year for black board features

Blackboard Tools	Year		
	2017 (*N* = 266)	2018 (*N* = 249)	2019 (*N* = 306)
Grade center	58 (21.8%)	101 (40.6%)	93 (30.4%)
Uploading and downloading	27 (10.2%)	04 (1.6%)	22 (7.2%)
Announcements	30 (11.3%)	03 (1.2%)	19 (6.2%)
Online quizzes	60 (22.6%)	43 (17.3%)	114 (37.3%)
Saudi Digital Library	38 (14.3%)	14 (5.6%)	24 (7.8%)

### Gender differences

Analysis of gender differences in the responses was conducted using independent sample T-Test. The results indicate instances of significant differences between male and female student responses. Table 5 shows instances of which responses are significantly different between genders. Positive t-values indicates significantly higher mean values for male students and vice versa.

**Table 5:** T-Test indicating differences in perceptions according to gender

Instances influenced by gender	t	df	Sig. (2-tailed)
The oral explanations in lectures helped me to understand textbook chapters	3.506	644	.000
I find weekly tutorial is a helpful learning activity.	4.753	653	.000
I find workshops are helpful learning activity	10.564	652	.000
Working in a group for workshops facilitated the learning process for me	5.957	643	.000
Reading, analyzing and writing report (i.e., Patient Safety case) helped to improve my analytical and writing skills	10.702	585	.000
Taking online quiz through Blackboard (LMS) is a good experience (convenient, enjoyable and easy)	−4.206	663	.000
I download content (slides, chapters, and papers) on Blackboard (LMS)	2.789	699	.005
I checked and received grade via Grade center on Blackboard (LMS)	−8.108	647	.000
The continuous oral and written feedback from course instructors through different channels (tutorial, workshop, quiz and exams) was effective in my learning	−2.943	656	.003
Class evaluation of presentations was effective	4.565	635	.000

### Academic years comparisons

Analysis of Variance (ANOVA) for statistical differences between the academic year was conducted to observe the differences between the medical years (Table 6). A statistically significant difference was found for 15 out of 22 items related to blended learning activities and learning questions. There are no statistically significant differences observed between preferred blended learning activity types nor between the years. The result is also the same for analysis on the years and the BB tools use. It was observed that the BB feature of “Online Quizzes” was the most preferable/useful tool in 2017, where the tool “Grade center” took most votes in 2018 and again was most voted in the year 2019.

**Table 6:** ANOVA that shows significant differences between years

Instances of significant difference between 2017, 2018 and 2019	F	Sig.
Lecture is a helpful learning activity	12.771	.000
The oral explanations in lectures helped me to understand textbook chapters	30.610	.000
I find workshops are helpful learning activity	38.311	.000
Working in a group for workshops facilitated the learning process for me	50.569	.000
Reading, analyzing and writing report (i.e. Patient Safety case) helped to improve my analytical and writing skills	15.848	.000
Mobile is a good experience (convenient, enjoyable and easy)	16.189	.000
Downloading lectures, reading materials and uploading assignments through Blackboard (LMS) facilitated my learning process	34.730	.000
Taking online quiz through Blackboard (LMS) is a good experience (convenient, enjoyable and easy)	15.735	.000
I download content (slides, chapters, and papers) on Blackboard (LMS)	33.588	.000
I checked and received grade via Grade center on Blackboard (LMS)	7.722	.000
The continuous oral and written feedback from course instructors through different channels (tutorial, workshop, quiz and exams) was effective in my learning	41.984	.000
Class evaluation of presentations was effective	28.097	.000
Using E-attendance was effective and save time during lectures, tutorials and workshops	60.518	.000
Compared to your other courses, was the workload in this course	21.979	.000
I would be interested to use Blackboard (LMS) in future training that is related to my medical education	7.144	.001

## Discussion

This research is highly essential in providing insights into how blended learning and modified TBL are perceived among medical students in flipped classroom environment. The three years cohort gather interesting findings such as gender differences in the perception of blended learning and variation of perception based on the different group by year cohort.

For gender analysis, it is found that there are significant differences in the t-test in most of the class activities. There are several class activities where male students have significant high mean values compared to the female counterpart which include oral explanations for understanding, tutorial and workshops as helpful learning activity, working in group, preparing report, downloading content from LMS and class evaluation on presentation. While female students have high mean values in the area of taking online quizzes, checking grades on LMS, and getting feedback from instructors.

In one recent study [9], male cohort group showed more satisfaction in blended learning than the female group due to several reasons which include computer self- efficacy factor. This makes sense as students acquire computer self-efficacy, they are comfortable to actively participate in blended learning activities. In our study, it can be seen that male students are also keen with the activities in the course in regard to blended learning activities. Similarly, another study by Dang et al. [27] informed that gender differences technology varies on how each gender utilize different features of the Internet. In agreement with our study results which showed female students used LMS features like quizzes and checking grades more than the male students group.

For the academic year’s comparisons across 2017,2018 and 2019, there are variation of preference on different blended learning strategies across the cohort group. We found that students in both 2018 and 2019 cohort were checking for grades from the LMS. In 2018 and 2019, our teaching team always reminded the students about their learning objectives, add orientation sessions on LMS and constant reminders via course announcements that may be a factor that create awareness among students to check their grades using LMS.

### Blended learning & modified team based learning

It is well understood that the course’s traditional nature is based on a highly practical method using face-to-face, field work and classroom activities. However, introducing blended learning is considered innovative yet challenging.

Therefore, investigating students’ perception can provide useful insights into how blended learning and modified TBL methods can be used effectively in other courses and to other groups of students. The blended learning approach was highly crucial in teaching medical informatics to overcome challenges faced due to a large number of students and the need for various exposures to reach the learning goals of the medical informatics course. On the overall, students who were taught using the flipped classroom environment have higher achievement levels compared to the traditional method. Similarly, students in our study showed a positive attitude towards flipped classroom activities. Students found tutorials and workshops to be effective learning activities and reported (agreed strongly and agreed) that working in groups for them (Tutorials: 53.3%; Workshops: 44.2%) had facilitated their learning process. More than half (51.6%) of the students correspond that team interactions in blended learning activities like tutorials and workshops have supported them to ask questions without feeling embarrassed. Research indicates that students generally prefer blended over face-to-face and fully online courses. A recent survey study by the EDUCAUSE Center for Applied Research of undergraduate students that most students prefer blended learning components over the four years the survey was done [28]. In a meta-analysis of 30 studies, Spanjers et al. [29] found that students slightly favored the blended learning approach. Our current study results show that students prefer blended learning activities, as they prefer face-to-face elements and online activities. About half of the students reported accessing the BB learning management system and download materials and articles; at the same time, more than 48% consider face-to-face lectures as helpful learning activities. Team-based learning has proven efficacy in different undergraduate courses in health sciences such as Nursing [30, 31], Pharmacy [31, 32], Speech Therapy [33], Medicine [34] and other specialties [35] . Due to time constraint and number of student in our Medical Informatics course, we were able to apply Modified TBL [36] instead the conventional TBL which enable one tutor to facilitate a number of groups simultaneously. For our class, students were in a group of 8–10 people throughout the one semester course. Pre-reading were required so that each group prepared the presentation materials. There were no readiness assurance testing process were given due to the time constraint and large number of students in the course. Following all of the modified TBL strategies, we obtained positive feedback whereby half of the students agreed that team interactions like in tutorials/workshops had allowed them to ask questions without feeling embarrassed. This encourage guided learning, problem solving, collaborative learning and critical reflection to happen naturally in the modified team based learning setting [36]. Collaborative learning occurred across this cohort as students felt working in team have facilitated their learning process while completing their pre reading and tasks before coming to tutorials and workshops. Most of the tasks require them to do problem solving before entering the sessions.

## Conclusion

Introducing blended learning and modified TBL is considered challenging. Therefore, the blended learning approach was highly important in teaching Medical Informatics subject to overcome challenges faced to due to the large number of students and the needs for various exposures to reach the learning goals of the medical informatics course. It was noticed that students preferred and were more engaged in both face-to-face and online activities. When comparing didactic teaching with that of online learning, using a LMS has facilitated the learning process, and e-learning can be viewed as a promising tool to improve the accessibility and availability of learning resources. The current study concluded as well that most of the medical students are satisfied with blended learning activities and that blended learning approaches were more helpful than both only traditional face-to-face learning and e-learning only approaches. Modified TBL encourages students to work conscientiously and effectively in teams. Students also reported being more comfortable working in teams and satisfied with the team members’ participation. Moreover, modified TBL was reported to enhance students’ understanding and improve their communication skills and self-confidence. The current study concluded a positive attitude towards flipped classroom activities as well. Students correspond that team interactions in blended learning activities like tutorials and workshops have supported them to ask questions without feeling embarrassed.

## Supplementary Information

**Additional file 1.**

## Data Availability

The datasets generated of the current study are not publicly available as student’s privacy could be compromised. However, it could be from the corresponding author on reasonable request.

## Abbreviations

CoM: College of Medicine
KSU: King Saud University
TBL: Team-based Learning
BB: BlackBoard
LMS: Learning Management System
IRAT: Individual Readiness Assurance Test
TRAT: Team Readiness Assurance Test
