# Supplementary material for: Modified team-based and blended learning perception: a cohort study among medical students at King Saud University
Source: BMC Med Educ. 2021 Apr 8;21:199. doi: 10.1186/s12909-021-02639-2 (PMC8034081; doi:10.1186/s12909-021-02639-2)
Supplement: Supplementary file 1 — Additional file 1. [file 12909_2021_2639_MOESM1_ESM.pdf]

Team-based and Blended Learning perception: A cohort study among medical students at King Saud University

Consent Form

Greetings Dear Students

We welcome you to participate in our study, which assesses team-based and blended learning perception among the students of medical students at College of Medicine, King Saud University (KSU).

Kindly take 5 - 8 minutes to answer this questionnaire, keeping in mind that your participation is entirely voluntary and confidential responses.

Thank you

I have read the consent form:

- ○ Yes, I ACCEPT to participate.
- ○ No, I do NOT ACCEPT to participate.

***Note:**

BL = Blended Learning

TL = Team-based Learning

Questionnaire

1. Which of the following academic activities is the most useful for you? **[BL]**
   A. Face to face lecture
   B. Discussions
   C. Workshops
   D. Tutorials
   E. Assignments

2. Lecture is a helpful learning activity. **[BL]**
   A. Strongly Agree
   B. Agree
   C. Undecided
   D. Disagree
   E. Strongly disagree

3. The oral explanations in lectures helped me to understand textbook chapters. **[BL]**
   A. Strongly Agree
   B. Agree
   C. Undecided
   D. Disagree
   E. Strongly disagree

4. I find weekly tutorial is a helpful learning activity. **[BL]**
   A. Strongly Agree
   B. Agree
   C. Undecided
   D. Disagree
   E. Strongly disagree

5. Working in a group for tutorial facilitated the learning process for me. **[TL]**
   A. Strongly Agree
   B. Agree
   C. Undecided
   D. Disagree
   E. Strongly disagree

6. I find workshops are helpful learning activity. **[BL]**
   A. Strongly Agree
   B. Agree
   C. Undecided
   D. Disagree
   E. Strongly disagree

7. Working in a group for workshops facilitated the learning process for me. **[TL]**
    A. Strongly Agree
    B. Agree
    C. Undecided
    D. Disagree
    E. Strongly disagree

8. Reading, analyzing and writing report (i.e., Patient Safety case) helped to improve my analytical and writing skills. **[BL]**
    A. Strongly Agree
    B. Agree
    C. Undecided
    D. Disagree
    E. Strongly disagree

9. Team interactions in tutorials/workshops allowed me to ask questions without feeling embarrassed. **[TL]**
    A. Strongly Agree
    B. Agree
    C. Undecided
    D. Disagree
    E. Strongly disagree

10. Using Blackboard (LMS) on mobile is a good experience (convenient, enjoyable and easy) **[BL]**
    A. Strongly Agree
    B. Agree
    C. Undecided
    D. Disagree
    E. Strongly disagree

11. I find Blackboard (LMS) is user friendly. **[BL]**
    A. Strongly Agree
    B. Agree
    C. Undecided
    D. Disagree
    E. Strongly disagree

12. Downloading lectures, reading materials and uploading assignments through Blackboard (LMS) facilitated my learning process. **[BL]**
    A. Strongly Agree
    B. Agree
    C. Undecided
    D. Disagree
    E. Strongly disagree

13. Taking online quiz through Blackboard (LMS) is a good experience (convenient, enjoyable and easy). **[BL]**
    A. Strongly Agree
    B. Agree
    C. Undecided
    D. Disagree
    E. Strongly disagree

14. I received email and read announcements from the Blackboard (LMS) for CMED 301 course updates. **[BL]**
    A. Always
    B. Frequently
    C. Sometimes
    D. Barely
    E. Never

15. I download content (slides, chapters, and papers) on Blackboard (LMS). **[BL]**
    A. Always
    B. Frequently
    C. Sometimes
    D. Barely
    E. Never

16. I checked and received grade via Grade center on Blackboard (LMS). **[BL]**
    A. Always
    B. Frequently
    C. Sometimes
    D. Barely
    E. Never

17. I access Saudi digital library through Blackboard (LMS) **[BL]**
    A. Always
    B. Frequently
    C. Sometimes
    D. Barely
    E. Never

18. I would be interested to use Blackboard (LMS) in future training that is related to my medical education. **[BL]**
    A. Always
    B. Frequently
    C. Sometimes
    D. Barely
    E. Never

19. Which of the following blackboard (LMS) tools is most useful? **[BL]**
    A. Grade Center
    B. Uploading and downloading
    C. Announcements
    D. Online quizzes
    E. Saudi Digital Library

20. Learning resources (slides, textbook chapters, published papers) facilitated my learning. **[BL]**
    A. Strongly Agree
    B. Agree
    C. Undecided
    D. Disagree
    E. Strongly disagree

21. Which learning resources you have used the most. **[BL]**
    A. Slides
    B. Textbook chapters
    C. Published papers
    D. Internet
    E. Tutorial resources

22. The continuous oral and written feedback from course instructors through different channels (tutorial, workshop, quiz and exams) was effective in my learning. **[BL]**
    A. Strongly Agree
    B. Agree
    C. Undecided
    D. Disagree
    E. Strongly disagree

23. Class evaluation of presentations was effective. **[BL]**
    A. Strongly Agree
    B. Agree
    C. Undecided
    D. Disagree
    E. Strongly disagree

24. Using E-attendance was effective and save time during lectures, tutorials and workshops. **[BL]**
    A. Strongly Agree
    B. Agree
    C. Undecided
    D. Disagree
    E. Strongly disagree

25. Compared to your other courses, was the workload in this course. **[BL]**
    A. Too Light
    B. Light
    C. Moderate
    D. Heavy
    E. Too Heavy

26. Gender
    A. Male
    B. Female

27. Academic Medical Year

    ____
